# Arthroscopic-assisted bone grafting and percutaneous K-wires fixation for the treatment of scaphoid nonunion in the skeletally immature patient: Three cases report

**DOI:** 10.1097/MD.0000000000024095

**Published:** 2021-01-22

**Authors:** Young-Keun Lee, Ki-Bum Kim

**Affiliations:** Department of Orthopedic surgery, Research Institute of Clinical Medicine of Jeonbuk National University-Biomedical Research Institute of Jeonbuk National University Hospital, Jeonbuk National University Medical School Jeonju, Jeonbuk, 54907 - 54896, Republic of Korea.

**Keywords:** arthroscopy, bone graft, K-wire, pediatric scaphoid nonunion

## Abstract

**Rationale:**

The treatment methods of pediatric scaphoid nonunion are still controversial. To our knowledge, arthroscopic-assisted treatments for pediatric scaphoid nonunion has not been reported in the English-language literature. Therefore, the purpose of this study is to report the use of arthroscopic-assisted bone grafting for scaphoid nonunion fracture in 3 patients and present a literature review.

**Patients concerns:**

Two 15-year-old patients developed carpal joint injuries over a year, prior to their hospital presentation, since they had not received adequate treatment. The third patient, 12 years of age, was diagnosed with scaphoid fracture after a traffic accident and underwent conservative treatment but presented to the hospital due to issues related with bone union.

**Diagnosis:**

All 3 patients were diagnosed with scaphoid nonunion at our hospital, using plain wrist radiographs and computed tomography.

**Interventions:**

All the patients underwent arthroscopic debridement; 2 patients received autogenous iliac cancellous bone graft, while the other patient received a bone substitute graft. The internal fixation of the scaphoid was performed with K-wires.

**Outcomes:**

Bone unions were achieved in all patients, and the final follow-up resulted in successful outcomes.

**Lessons:**

Arthroscopic-assisted bone grafting and percutaneous K-wire fixation can be considered as a good method for the treatment of pediatric scaphoid nonunion fractures. Therefore, it is a primary treatment option for symptomatic scaphoid nonunion fracture and displaced fractures.

## 1 Introduction

Scaphoid nonunion in children, is a rare complication representing as low as 0.8% of the scaphoid fracture.^[1]^ Due to the rarity of this condition, a natural history has not been established in contrast to the adults, and it cannot be established to what extent a pediatric scaphoid nonunion should be treated. Therefore the optimal treatment of a pediatric scaphoid nonunion remains controversial.^[2–5]^

Recently, positive results have been reported from conservative treatment of proximal pole nonunion.^[6,7]^ However, the prolonged treatment time has been considered a disadvantage. On the other hand, surgical treatment has the advantage of achieving early bone union with fracture reduction and the ability to return to daily life faster.

The arthroscopic-assisted bone grafting method has recently been introduced for surgical treatment of scaphoid nonunion. This method, being less invasive in bone grafting at the nonunion site, enables the simultaneous diagnosis and treatment of associated injuries. Considering the early bone union, it is reported as a treatment method with the advantages of faster rehabilitation and hand function.^[8,9]^ However, there have been no reports of arthroscopic-assisted treatments in pediatric scaphoid nonunion treatments. Therefore, the purpose of this study is to report 3 cases of arthroscopic-assisted bone grafting treatment for scaphoid nonunion fractures with literature reviews.

### 1.1 Consent

The patient signed an informed consent for the publication of this case report and any accompanying images. We obtained parent or guardian consent to publish our findings. The ethical approval of this study was waived by the ethics committee of Jeonbuk National University Hospital because it was a case report and there were fewer than 3 patients.

## 2 Methods

### 2.1 Patients

A total of 52 patients underwent arthroscopic bone grafting and Kirschner (K)-wires fixation to treat scaphoid nonunion at our institution between November 2008 and November 2019. The inclusion criteria of patients for this cases report was skeletally immature patients who received arthroscopic bone grafting and K-wires fixation to treat scaphoid nonunion. Skeletal immaturity was defined as open growth plates in both distal radius and ulna at the time of operation. The patients included 3 males with an average age of 14 years (range, 12–15 years). The time interval between injury and operation ranged from 3 to 18 months (average 11 months).

### 2.2 Surgical procedure

The operation is performed under general anesthesia and the patient is positioned supine with the contralateral side of the iliac crest region draped for bone graft harvesting as described in previous the studies.^[8,9]^ The operated arm is placed in a wrist traction tower and a vertical traction of 4 to 6 kg force is applied through plastic finger trap devices to the middle 3 fingers for joint distraction on a hand table. An arm tourniquet is not applied and a C-arm image intensifier is prepared for the percutaneous scaphoid fracture reduction and K-wires fixation.

We use a 2.5 mm video arthroscope (Linvatec, CONMED Linvatec, Utica, NY), 2.0 mm and 2.9 mm shavers, a 3.0 mm burr, and a radiofrequency probe for surgical instruments. We also use 2 custom made cannulas (3.8 mm and 3.0 mm) and 2 custom made trocars (3.2 mm and 2.7 mm) for percutaneous bone grafting. We employ continuous sending irrigation. We make 3/4 and 4/5 portals for the radio-carpal joint, and mid-carpal radial (MCR), mid-carpal ulna, and 1 accessory portal for the mid-carpal joint.

We perform inspection on the radio-carpal joint at first. During arthroscopy, particular attention is paid to observe the status of the interosseous ligaments, articular cartilage, the presence of synovitis. We then transfer the arthroscope to the mid-carpal joint and examine the status of the articular cartilage and nonunion site. Both ends of the nonunion site are debrided and burred by switching the burr and shaver to the MCR and accessory portals until healthy looking cancellous bone with punctate bleeding can be seen. After preparing the bone graft, we reduce the scaphoid under the C-arm image intensifier and a 1.2 mm K-wire is inserted percutaneously from the tubercle of the scaphoid to the proximal pole for provisional scaphoid fixation. In the presence of a dorsal intercalated segmental instability deformity and extended lunate, we first flex the wrist to realign the extended lunate with the radius for deformity correction. The radio-lunate joint is then transfixed with a percutaneous 1.2 mm K-wire inserted from the dorsal distal radius. We then percutaneously fix the scaphoid with a 1.2 mm K-wire. We confirm the position of the K-wire under arthroscopic view before proceeding to the bone graft. Cancellous bone graft is harvested from the iliac crest instead of distal radius using an open approach through a small incision. We want to get the volume of the harvested bone graft that has to be at least 3 to 5 times that of the defect because the graft needs to be tightly compressed into the defect to increase the strength of the graft. The bone graft is then cut into small chips using scissors. In 1 of the 3 patients, a bone substitute graft (Genesis TM Sponge, Seoul, Hanmi, Korea) was used instead of the autogenous iliac cancellous bone.

For bone grafting, an arthroscope is introduced in the MCU portal to continuously show the nonunion site, a custom made 3.8 mm cannula is introduced to the nonunion site through the MCR portal, and cancellous chip bone and bone substitute graft were delivered to the entrance of the cannula. The bone graft is impacted with 3.2 mm trocar until a satisfactory volume of graft is achieved. After completely filling the defect, routine surveillance of the joint is carried out to detect and remove any spilled bone graft material. We then routinely inject 1 cc of fibrin glue (Greenplast Kit, Green Cross,Yongin Korea) into the surface of the graft substance. After arthroscopy, the wrist is taken out of traction. Definitive fixation with 2 1.2 mm K-wires is performed under the C-arm image intensifier. Additional scapholunate (SL) K-wires fixation is performed to fix the unstable nonunion and is kept in place for 8 weeks. K-wires are then placed outside the skin.

Postoperatively, the wrist is immobilized with an above elbow thumb spica splint for 2 weeks due to the protection of RL pinning which was removed at 2 weeks, after which we apply the below elbow thumb spica cast for 8 weeks. Plain radiographs are taken every week until bone union is achieved. When radiological union is confirmed, the K-wires are removed.

### 2.3 Radiographic and clinical evaluation

Bone union was clinically assessed as the absence of tenderness at the anatomical snuffbox and radiologically with wrist posteroanterior (PA), lateral, semipronated oblique, and PA with ulnar deviation view assessed as the disappearance of the fracture line with bony trabecular across the original fracture. Patients were evaluated for range of motion (ROM), and grip strength measured on a dynamometer.^[10]^ Functional outcome was evaluated from a comparison between the Quick DASH (disabilities of the arm, shoulder, and hand) and the visual analogue scale (VAS) for pain (0 = no pain, 10 = worst pain), which were measured at the time of preoperation and final follow up.^[11,12]^

## 3 Case presentation

### 3.1 Case 1

A 15-year-old boy presented to the clinic with a right wrist pain, which is aggravated for 3 months ago. The patient hurt his right wrist after a fall while playing soccer a year prior to the presentation. The patient did not receive any treatment despite experiencing pain during laborious tasks, since he was able to perform daily activities. Upon physical examination, the radial side of the wrist had mild edema and evident anatomical snuff box tenderness. The patient displayed a limited ROM with flexion and extension angles of 70° and 60°, respectively, in the right wrist (80° and 90° in the left wrist, respectively). The radial and ulnar deviation angles in the right wrist were 15° and 50°, respectively (25° and 50° in the left wrist, respectively). When we examined the patient, the wrist showed mild swelling on the radial aspect as well as anatomical snuff box tenderness. He displayed limited ROM, flexion and extension angles of the right wrist were 70° and 60°, respectively (with 80° and 90° in the left). The radial and ulnar deviation angles in the right wrist were 15° and 50°, respectively (with 25° and 50° in the left).

Initial plain wrist radiographs demonstrated nonunion of a fracture in the waist of the scaphoid (Fig. 1  A). A computed tomography (CT) was obtained and indicated nonunion in the scaphoid waist and lateral intra-scaphoid angle of about 88°. The results induced the finding of humpback deformity (Fig. 1  B).

**Figure 1:**
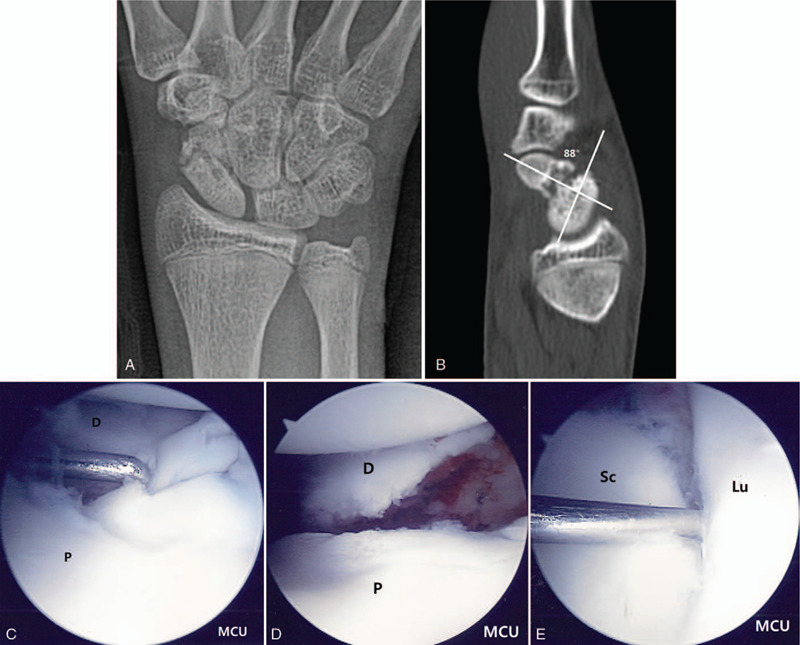
A 15-year-old boy patient with nonunion of the right scaphoid fracture. (A) Preoperative right wrist plain PA with ulnar deviation view showing nonunion at the waist of the scaphoid. (B) Preoperative right wrist sagittal CT scan shows nonunion at the waist of the scaphoid, about 88 degrees lateral intra-scaphoid angle and a humpback deformity. (C) Mid-carpal arthroscopy image of scaphoid nonunion site (P: proximal fragment, D: distal fragment). (D) Mid-carpal arthroscopy image of nonunion site after debridement showing punctate bleeding from proximal and distal fragments. (E) Mid-carpal arthroscopy image of SL joint showing volar side instability (Sc: scaphoid, Lu: lunate). (F) A mid-carpal arthroscopy image of percutaneous autogenous iliac cancellous bone grafting at the nonunion site using cannula and trocar (left) and complete the bone graft (right). (G) Immediate postoperative plain right wrist PA with ulnar deviation and lateral radiographs show internal fixation with K-wires and grafted bone at the nonunion site. (H) Postoperative 9 weeks K-wires removal axial and sagittal CT scans show complete bony union and correction of humpback deformity. (I) At 54 months’ follow-up plain right wrist PA with ulnar deviation and lateral radiographs show solid bony union.

**Figure 1 (Continued):**
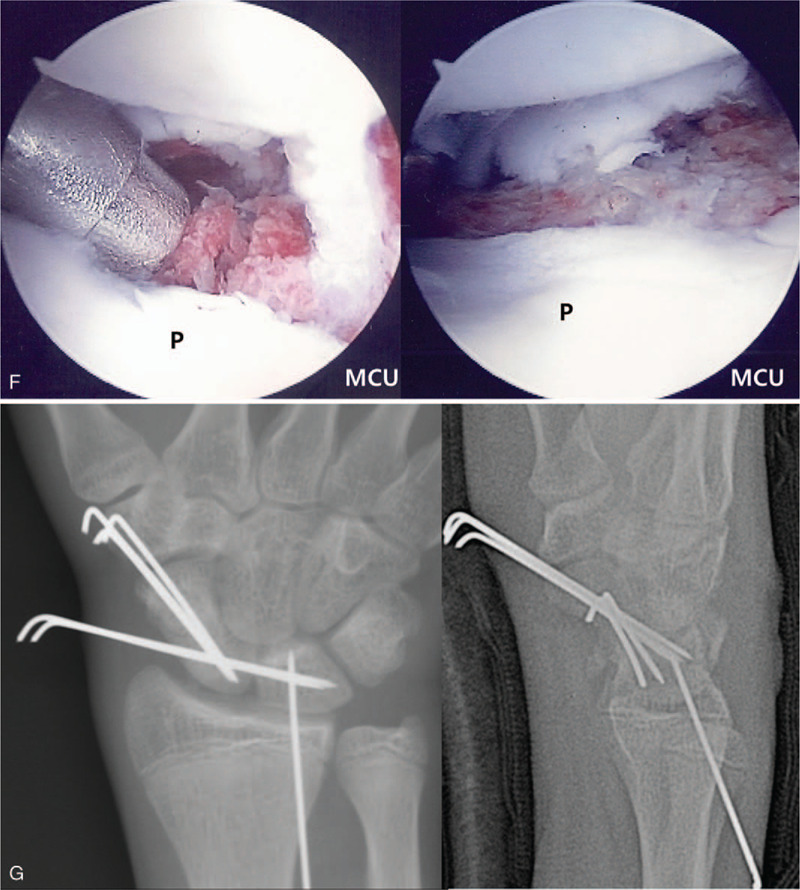
A 15-year-old boy patient with nonunion of the right scaphoid fracture. (A) Preoperative right wrist plain PA with ulnar deviation view showing nonunion at the waist of the scaphoid. (B) Preoperative right wrist sagittal CT scan shows nonunion at the waist of the scaphoid, about 88 degrees lateral intra-scaphoid angle and a humpback deformity. (C) Mid-carpal arthroscopy image of scaphoid nonunion site (P: proximal fragment, D: distal fragment). (D) Mid-carpal arthroscopy image of nonunion site after debridement showing punctate bleeding from proximal and distal fragments. (E) Mid-carpal arthroscopy image of SL joint showing volar side instability (Sc: scaphoid, Lu: lunate). (F) A mid-carpal arthroscopy image of percutaneous autogenous iliac cancellous bone grafting at the nonunion site using cannula and trocar (left) and complete the bone graft (right). (G) Immediate postoperative plain right wrist PA with ulnar deviation and lateral radiographs show internal fixation with K-wires and grafted bone at the nonunion site. (H) Postoperative 9 weeks K-wires removal axial and sagittal CT scans show complete bony union and correction of humpback deformity. (I) At 54 months’ follow-up plain right wrist PA with ulnar deviation and lateral radiographs show solid bony union.

**Figure 1 (Continued):**
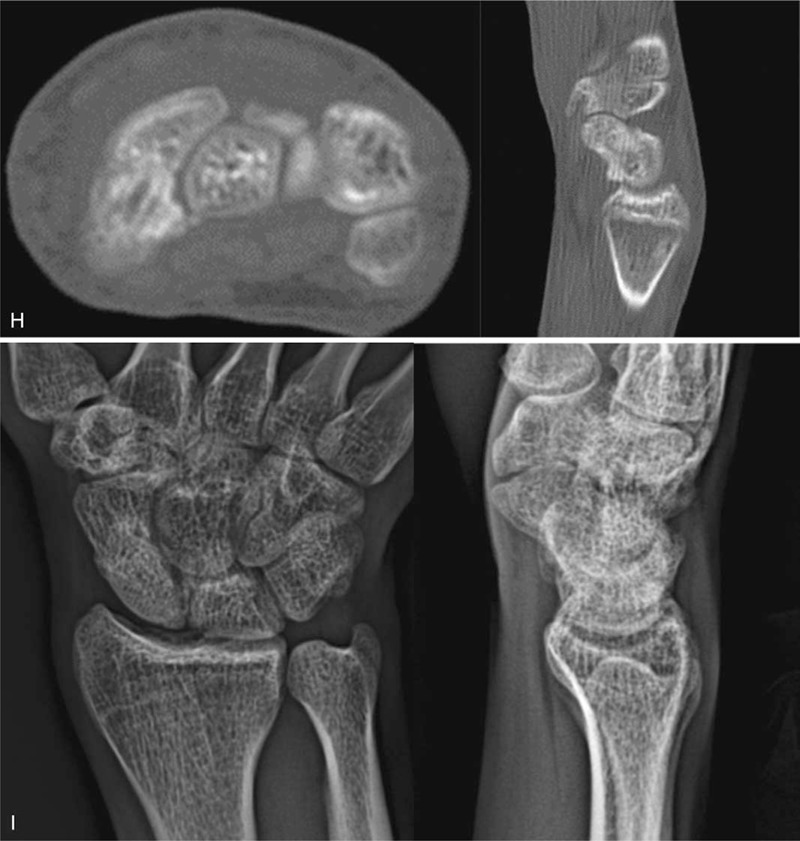
A 15-year-old boy patient with nonunion of the right scaphoid fracture. (A) Preoperative right wrist plain PA with ulnar deviation view showing nonunion at the waist of the scaphoid. (B) Preoperative right wrist sagittal CT scan shows nonunion at the waist of the scaphoid, about 88 degrees lateral intra-scaphoid angle and a humpback deformity. (C) Mid-carpal arthroscopy image of scaphoid nonunion site (P: proximal fragment, D: distal fragment). (D) Mid-carpal arthroscopy image of nonunion site after debridement showing punctate bleeding from proximal and distal fragments. (E) Mid-carpal arthroscopy image of SL joint showing volar side instability (Sc: scaphoid, Lu: lunate). (F) A mid-carpal arthroscopy image of percutaneous autogenous iliac cancellous bone grafting at the nonunion site using cannula and trocar (left) and complete the bone graft (right). (G) Immediate postoperative plain right wrist PA with ulnar deviation and lateral radiographs show internal fixation with K-wires and grafted bone at the nonunion site. (H) Postoperative 9 weeks K-wires removal axial and sagittal CT scans show complete bony union and correction of humpback deformity. (I) At 54 months’ follow-up plain right wrist PA with ulnar deviation and lateral radiographs show solid bony union.

Arthroscopic bone grafting and percutaneous K wires fixation was performed under general anesthesia. The mid-carpal joint arthroscopic findings revealed nonunion in the waist area, with punctate bleeding observed in proximal and distal fragments after arthroscopic debridement and removal of necrotic tissue, at the nonunion site. The associated injury was Geissler grade II instability at the volar side of the SL joint (Fig. 1  C-G).^[13]^ We could get a complete bone union at 9 weeks after operation (Fig. 1  H).

At 54 months’ follow-up, the preoperative pain of the patient improved on the VAS from 3 to 0, and the preoperative Quick DASH score also improved from 29.5 to 0. The ROM and grip strength improved from the preoperative normal range of 80% to 95% and 50% to 84%, respectively (Fig. 1  I).

### 3.2 Case 2

A 15-year-old boy presented with pain in the right wrist, which was reported to worsen during flexion and extension of the wrist. He was diagnosed with right scaphoid fracture after hitting a wall in school, 1 year and 6 months prior to the presentation. He was treated with a splint, but he removed the splint on his own after 10 days. Afterwards, he received no other treatment.

The physical examination showed no swelling in the right wrist but tenderness in the radial side. The ROM was as follow: 45° in flexion, 45° in extension, 20° in radial deviation, and 35° in ulnar deviation, 85.3% of normal side. The VAS score for pain was 7.

**Figure 2:**
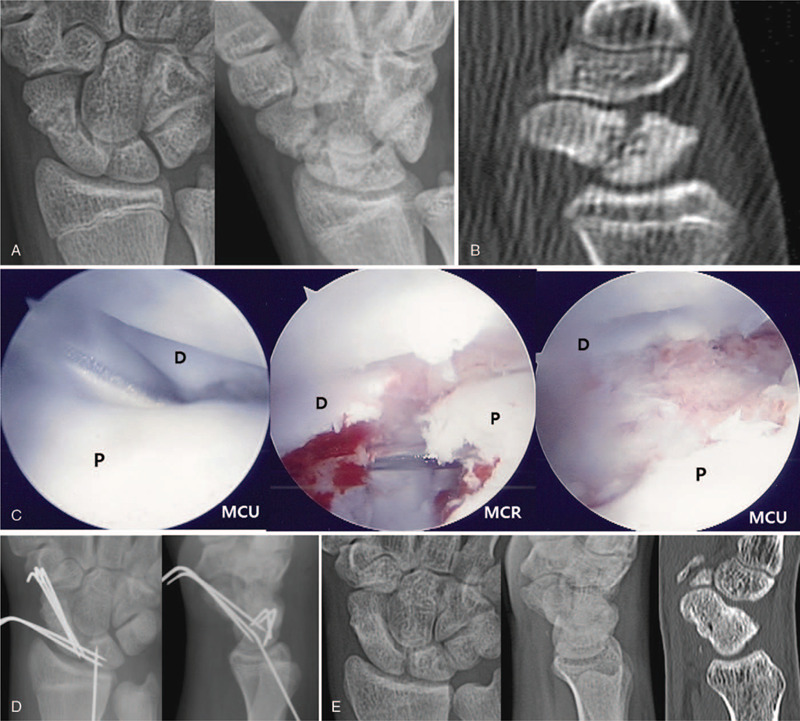
A 15-year-old boy patient with nonunion of the right scaphoid fracture (A) Preoperative right wrist plain PA with ulnar deviation and oblique view showing nonunion at the waist of the scaphoid. (B) Preoperative right wrist sagittal CT scan shows nonunion at the waist of the scaphoid. (C) Intraoperative mid-carpal arthroscopy image of scaphoid nonunion site showing the gap at the waist of the scaphoid (left, P: proximal fragment, D: distal fragment), provisional K-wire fixation (center) and grafted iliac cancellous bone at the nonunion site (right). (D) Immediate postoperative plain right wrist PA and lateral radiographs show internal fixation with K-wires and grafted bone at the nonunion site. (E) At 54 months’ follow-up plain right wrist PA with ulnar deviation, lateral radiographs and sagittal CT scan show sold bony union.

On the initial plain radiographs, a nonunion was identified at the waist of the scaphoid (Fig. 2A, B). Arthroscopic-assisted bone grafting was performed. Arthroscopic findings revealed nonunion at the waist, and punctate bleeding was found in both bone fragments after debridement. Associated injuries were identified to be Geissler grade II SL instability (Fig. 2C). Definitive fixation was performed using K-wires (Fig. 2D). The bone union has been obtained 10 weeks after operation.

At 54 months follow-up, a solid bone union was achieved on plain radiograph (Fig. 2E). VAS score was 2.8, Quick DASH score improved to 9.1, and the ROM improved to the normal range of 92.5%.

### 3.3 Case 3

A 12-year-old boy presented with a left scaphoid nonunion fracture. Three months prior to the hospital visit, he was diagnosed with left scaphoid fracture and received conservative treatment, but no bone union was observed at other hospital.

There was no pain in the left wrist at the time of admission, and the patient had short arm thumb spica cast. Initial plain radiographs and CT demonstrated nonunion of a fracture in the distal part of the scaphoid (Fig. 3 A, B). Arthroscopic–assisted bone grafting was planned. The mid-carpal joint arthroscopic findings showed fibrotic tissue covering the section of the scaphoid fracture with no sclerotic bone findings. In addition, the SL ligament showed Geissler grade II instability. The fibrotic tissue at the fracture site was removed with a shaver and punctate bleeding was confirmed (Fig. 3 C). Provisional fixation was performed using 1.2 mm K-wire, and bone substitute graft (Genesis TM Sponge, Seoul, Hanmi, Korea) was used instead of the autogenous iliac cancellous bone (Fig. 3 D). Thermal shrinkage was performed for SL ligament instability. Definitive fixation was performed using K-wires (Fig. 3 E).

**Figure 3:**
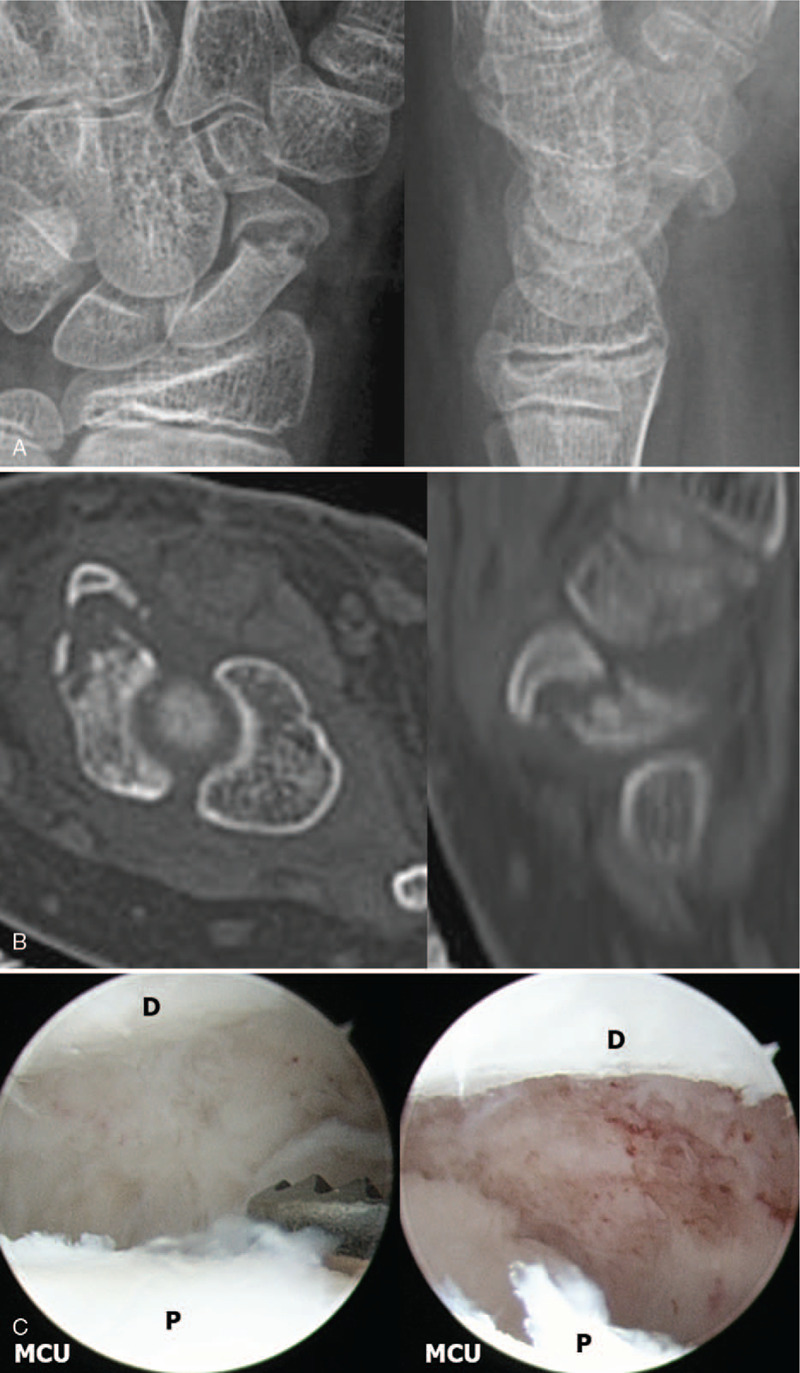
An 11-year-old boy patient with nonunion of the left scaphoid fracture. (A) preoperative left wrist plain PA with ulnar deviation and lateral views show nonunion at the distal pole of the scaphoid. (B) Preoperative left wrist axial and sagittal CT scan show nonunion and bone defect at the distal pole of the scaphoid. (C) Mid-carpal arthroscopy image of scaphoid nonunion site showing the gap at the distal pole of the scaphoid and it covered with fibrotic tissue (left, P: proximal fragment, D: distal fragment) after debridement of fibrotic tissue at the ends of fragments showing punctate bleeding from the distal fragment (right). (D) Arthroscopy image after Genesis sponge grafting at the nonunion site. (E) Immediate postoperative plain left wrist PA with ulnar deviation and lateral radiographs show well reduced scaphoid fracture and internal fixation with K-wires. (F) At 34 months’ follow-up plain left wrist PA with ulnar deviation, lateral radiographs and clinical photos show solid bone union and excellent functional results.

**Figure 3 (Continued):**
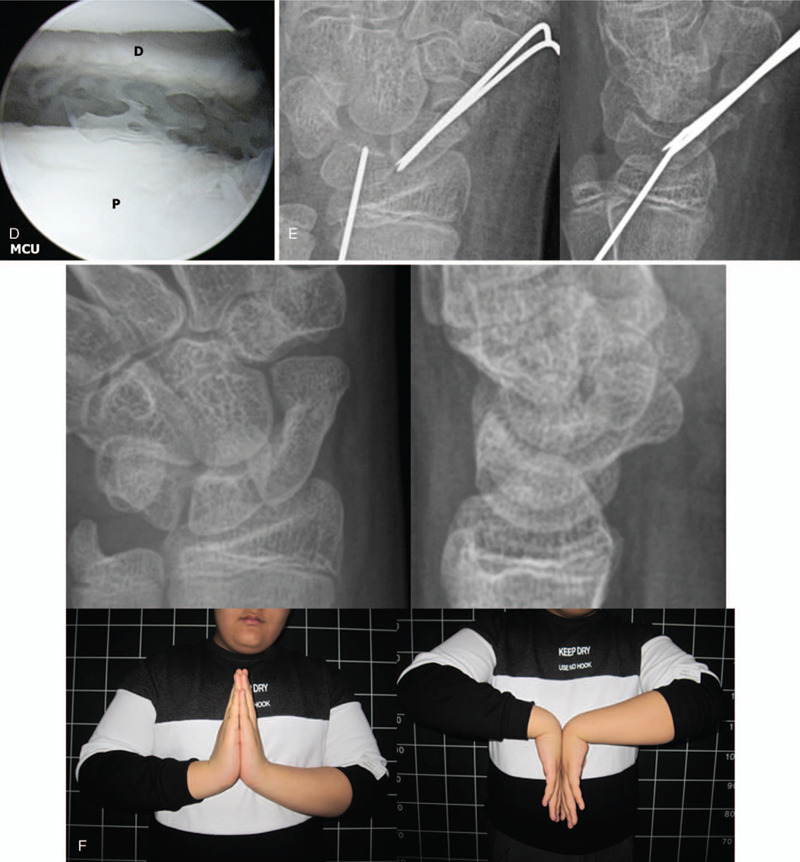
An 11-year-old boy patient with nonunion of the left scaphoid fracture. (A) preoperative left wrist plain PA with ulnar deviation and lateral views show nonunion at the distal pole of the scaphoid. (B) Preoperative left wrist axial and sagittal CT scan show nonunion and bone defect at the distal pole of the scaphoid. (C) Mid-carpal arthroscopy image of scaphoid nonunion site showing the gap at the distal pole of the scaphoid and it covered with fibrotic tissue (left, P: proximal fragment, D: distal fragment) after debridement of fibrotic tissue at the ends of fragments showing punctate bleeding from the distal fragment (right). (D) Arthroscopy image after Genesis sponge grafting at the nonunion site. (E) Immediate postoperative plain left wrist PA with ulnar deviation and lateral radiographs show well reduced scaphoid fracture and internal fixation with K-wires. (F) At 34 months’ follow-up plain left wrist PA with ulnar deviation, lateral radiographs and clinical photos show solid bone union and excellent functional results.

Bone union was confirmed 3 months after the operation.

At 34 months follow-up, complete bone union was obtained. There was no pain and the ROM was in the normal range with 96%, grip strength was 69%, and the Quick DASH score improved from 63.8 preoperatively to 6.8 last follow up (Fig. 3 F).

## 4 Discussion

Until now, the treatment of pediatric scaphoid nonunion is controversial owing to the rarity of this injury pattern. Some authors advocate early surgical treatment and others recommend an initial trial of nonsurgical treatment.^[2–7,14]^ However, with the recent increase in incidence of scaphoid fractures in children, the incidence of nonunion is also expected to increase.^[15]^ Thus, we believe that it is necessary to establish certain treatment outlines. Though conservative treatment can be considered reasonable, surgical method is advantageous since pediatric scaphoid in children is fundamentally different to that of adults, with continuous evolution of the physical properties of the scaphoid during the maturation period (6 – 15 years).^[16]^ Recently, conservative treatment has been reported to be beneficial for difficult to treat proximal pole nonunion with avascular necrosis (AVN).^[6,7]^ However, there are disadvantages, which include the prolonged treatment time and the anxiety encountered by the parents during the selection process between surgical or conservative treatment. In addition, the diagnosis of AVN based on magnetic resonance imaging alone as described by Jermigan et al^[6]^, is difficult to an accuracy of 68% to 78%. According to Green's prone viability test, the diagnosis was confirmed as AVN in the absence of punctate bleeding.^[17]^ Therefore, the authors believe that even in pediatric scaphoid nonunion fractures, surgical treatment are necessary in symptomatic nonunion and displaced fractures, just like the treatments for adult scaphoid nonunion fractures. All of our cases were symptomatic and had displaced nonunion fractures.

Though surgical methods for scaphoid nonunion have been in use, a bone grafting method using arthroscopy, which has advantages over the open surgery methods, has been recently introduced.^[8,9,18]^ Chu and Shih,^[18]^ also reported positive results from implanting an injectable bone graft substitute instead of autogenous cancellous bone for early stage scaphoid nonunion. Therefore, the authors undertook a less invasive arthroscopic-assisted bone grafting rather than the conservative treatment for symptomatic pediatric scaphoid nonunion patients. No significant difference were found between the generation of the portal and instrument preparation in the surgical method.^[9]^ However, due to thinner fingers, a thin layer of self-adhesive wrap (3 M^TM^ Coban^TM^ band, St. Paul, MN) was applied to equip the patient with a finger trap. Two patients underwent autogenous iliac cancellous bone graft after nonunion site debridement, and 1 patient had early stage nonunion in which punctate bleeding was confirmed by removing the thin layer of fibrotic tissue covering the nonunion with a shaver. Therefore, grafting was attempted using bone graft substitutes as suggested by Chu and Shih.^[18]^

The advantages of arthroscopic assisted percutaneous and minimally invasive procedures can (1) avoid carpal ligament injury because they do not require an open arthrotomy and can preserve as much of the tenuous blood supply of scaphoid as possible.^[8,9]^ The minimal disturbance of the vascularity of the scaphoid may contribute to the high union rate and a relatively short period for bone union compared to open treatment. The authors were fortunate to obtain bone union in the 3 cases at 9, 10, and 12 weeks, respectively. Furthermore, although carpal ligament injury was present in all the cases, 1 case had accompanying humpback deformity, however, the ROM of patients at the final follow up had improved in all cases. We believe this is because we avoided additional injury of the joint capsule and carpal ligament through the minimally invasive method. (2) Accurate diagnosis and treatment for intra-articular pathology and cartilage are possible at the same time. We identified SL ligament injuries in all patients. All 3 cases showed Geissler grade II instability and therefore, thermal shrinkage was performed.^[10]^

Methods of internal fixation have evolved over time. Since the introduction of the Herbert screw (which provides compression force and rigidity at the nonunion site), its success has resulted in further developments in headless screws, which have compressive abilities and have recently been widely used.^[19,20]^ However, whether this compression enhances healing at the nonunion site remains unknown.^[21]^ Nevertheless, screw fixation has a number of disadvantages such as the need to drill along the scaphoid central axis without loss of alignment, difficulty in determining the length and diameter of the screw, potential collapse of corticocancellous bone at the shank of the screw, risk of the screw remaining in the body permanently and additional constructs are required if rigid fixation cannot be obtained by screw fixation alone. Therefore, screw fixation is a riskier technique than K-wires fixation. K-wires provide rigidity and resistance to bending but no compression force across the fracture site compared to screws. In addition, the lack of compression force can be solved by sufficient bone grafting. We used K-wires for fixation of scaphoid. Chen et al^[22]^ reported a 100% union rate in 39 patients treated for scaphoid nonunion with corticocancellous bone graft and multiple, divergent K-wire fixation. Takami et al^[23]^ reported a 98% union rate in 43 patients using corticocancellous bone graft combined with K-wire fixation. The authors obtained bone union without complications in all cases. It has advantages such as a greater ease of insertion, a shorter operative time, a minimum amount of dissection. Moreover, the removal is easy after the bone union. In our cases, it was possible to perform removal after the union in the outpatient department. In addition, if we cannot achieve bone union, the K-wire is a more useful technique in the preparing for the secondary operation.

Our study has a number of limitations. First, a small number of patients were included. Due to the lack of pediatric scaphoid fractures, it is a pity that reports were produced in the case report despite the applying the new surgical method. Secondly, it is difficult to directly compare the results of the authors with the treatment results using open methods. Thirdly, the observers were not independent from the clinical and radiographic outcomes.

## 5 Conclusion

Arthroscopic- assisted bone grafting and percutaneous K-wires fixation may be a good treatment method for pediatric scaphoid nonunion. Therefore, it can be selected as a primary treatment method to treat pediatric symptomatic scaphoid nonunion and displaced fractures.

## Author contributions

**Conceptualization:** Young Keun Lee, Ki-Bum Kim.

**Data curation:** Young Keun Lee.

**Formal analysis:** Young Keun Lee.

**Funding acquisition:** Young Keun Lee.

**Investigation:** Young Keun Lee.

**Methodology:** Young Keun Lee, Ki-Bum Kim.

**Project administration:** Young Keun Lee.

**Resources:** Young Keun Lee.

**Software:** Young Keun Lee.

**Supervision:** Young Keun Lee.

**Validation:** Young Keun Lee.

**Visualization:** Young Keun Lee.

**Writing – original draft:** Young Keun Lee.

**Writing – review & editing:** Young Keun Lee, Ki-Bum Kim.

## References

[1] Fabre O De Boek H Haentjens P Fractures and nonunion of the carpal scaphoid in children. Acta Orthop Belg 2001;76:121–5. 11383289

[2] Chloros GD Themistocleous GS Wiesler ER Pediatric scaphoid nonunion. J Hand Surg Am 2007;32:172–6. 17275591 10.1016/j.jhsa.2006.11.007

[3] Duteille F Dautel G Nonunion fractures of the scaphoid and carpal bones in children: surgical treatment. J Pediatr Orthop 2004;13:34–8. 10.1097/00009957-200401000-00007 15091257

[4] Garcia-Mata S Carpal scaphoid fracture nonunion in children. J Pediatric Orthop 2002;22:448–51. 12131439

[5] Anz AW Bushnell BD Bynum DK Pediatric scaphoid fracture. J Am Acad Orthop surg 2009;17:77–87. 19202121 10.5435/00124635-200902000-00004

[6] Jernigan EW Smetana BS Patterson MM Pediatric scaphoid proximal pole nonunion with avascular necrosis. J Hand Surg Am 2017;42:299.e1–4. 28027846 10.1016/j.jhsa.2016.11.018

[7] Rupani N Riley N Mcnab I Spontaneous healing of a pediatric scaphoid proximal pole fracture nonunion. J Wrist Surg 2018;7:81–3. 29383281 10.1055/s-0037-1602799 PMC5788760

[8] Wong WC Ho PC Arthroscopic management of scaphoid nonunion. Hand Clin 2019;35:295–313. 31178088 10.1016/j.hcl.2019.03.003

[9] Lee YK Choi KW Woo SH The clinical result of arthroscopic bone grafting and percutaneous K-wires fixation for management of scaphoid nonunion. Medicine (Baltimore) 2018;97:e9987. 29595703 10.1097/MD.0000000000009987 PMC5895373

[10] Angst F Drerup S Werle S Prediction of grip and key pinch strength in 978 healthy subjects. BMC Musculoskelet Disord 2010;11:94. 20482832 10.1186/1471-2474-11-94 PMC2882344

[11] Cooney WP 3rd Cooney WP 3rd Wrist scoring system and clinical assessment. The wrist: diagnosis and operative treatment. 2nd ed. Philadelphia: Lippincott Williams & Wilkins; 2010 205–14.

[12] Beaton DE Wright JG Katz JN Upper extremity collaborative group. Development of the Quick DASH: comparison of three item-reduction approaches. J Bone Joint Surg Am 2005;87:1038–46. 15866967 10.2106/JBJS.D.02060

[13] Geissler WB Freeland AE Savoie FH Intracarpal soft-tissue lesions associated with an intra-articular fracture of the distal end of the radius. J Bone Joint Surg Am 1996;78:357–65. 8613442 10.2106/00004623-199603000-00006

[14] Henderson B Lett M Operative management of pediatric scaphoid fracture nonunion. J Pediatr Orthop 2003;23:402–6. 12724610

[15] Ahmed I Ashton F Tay WK The pediatric fracture of the scaphoid in patients aged 13 years and under: an epidermiological study. J Pediatr Orthop 2014;34:150–4. 24172664 10.1097/BPO.0000000000000102

[16] Chloros GD Kelalis GH Wiesler ER Slutsky DJ, Slade JF 3rd Pediatric scaphoid fractures and nonunion. The scaphoid.. New York, NY: Thieme; 2011 204–16.

[17] Green DP The effect of avascular necrosis on Russe bone grafting for scaphoid nonunion. J Hand Surg Am 1985;10:597–605. 3900189 10.1016/s0363-5023(85)80191-x

[18] Chu PJ Shih JT Arthroscopically assisted use of injectable bone graft substitutes for management of scaphoid nonunions. Arthroscopy 2011;27:31–7. 20934844 10.1016/j.arthro.2010.05.015

[19] Capo JT Orillaza NS Jr Slade JF 3rd Percutaneous management of scaphoid nonunions. Tech Hand Up Extrem Surg 2009;13:23–9. 19276923 10.1097/BTH.0b013e3181877644

[20] Slade JF 3rd Merrell GA Geissler WB Geissler WB Fixation of acute and selected nonunion scaphoid fractures. Wrist arthroscopy.. New York, NY: Springer; 2005 112–24.

[21] Trail IA Stanley JK Slutsky DJ Scaphoid nonunions: predictive factors. Principles and practice of wrist surgery.. Philadelphia: Elsevier; 2010 233–8.

[22] Chen CY Chao EK Lee SS Osteosynthesis of carpal scaphoid nonunion with interpositional bone graft and Kirschner wires: a 3-to 6-year follow-up. J Trauma 1999;47:558–63. 10498315 10.1097/00005373-199909000-00024

[23] Takami H Takahashi S Ando M Scaphoid nonunion treated by open reduction, anterior inlay bone grafting, and Kirschner-wire fixation. Arch Orthop Trauma Surg 2000;120:134–8. 10738869 10.1007/pl00013760

